# A mixed distribution to fix the threshold for Peak-Over-Threshold wave height estimation

**DOI:** 10.1038/s41598-022-22243-8

**Published:** 2022-10-15

**Authors:** Antonio M. Durán-Rosal, Mariano Carbonero, Pedro Antonio Gutiérrez, César Hervás-Martínez

**Affiliations:** 1 Department of Quantitative Methods, Universidad Loyola Andalucía, Córdoba, Spain; 2 Department of Computer Science and Numerical Analysis, University of Córdoba, Córdoba, Spain

**Keywords:** Physical oceanography, Ocean sciences

## Abstract

Modelling extreme values distributions, such as wave height time series where the higher waves are much less frequent than the lower ones, has been tackled from the point of view of the Peak-Over-Threshold (POT) methodologies, where modelling is based on those values higher than a threshold. This threshold is usually predefined by the user, while the rest of values are ignored. In this paper, we propose a new method to estimate the distribution of the complete time series, including both extreme and regular values. This methodology assumes that extreme values time series can be modelled by a normal distribution in a combination of a uniform one. The resulting theoretical distribution is then used to fix the threshold for the POT methodology. The methodology is tested in nine real-world time series collected in the Gulf of Alaska, Puerto Rico and Gibraltar (Spain), which are provided by the National Data Buoy Center (USA) and Puertos del Estado (Spain). By using the Kolmogorov-Smirnov statistical test, the results confirm that the time series can be modelled with this type of mixed distribution. Based on this, the return values and the confidence intervals for wave height in different periods of time are also calculated.

## Introduction

Marine forecasting has become a essential task to ensure the safety of navigation, fishery and engineering construction, among others^1^. Concretely, wave height prediction is key to the design of coastal and off-shore structures^2^. In this sense, the incorporation of wave models into numerical weather prediction models can improve atmospheric forecasts^3^. The development of offshore installations for oil and gas extraction and for renewable energy exploitation requires knowledge of the wave fields and any potential changes in them. One of the main problems is that the knowledge of the maximum peak-to-trough wave height is not usually available although largest waves have the greatest impact on ships and offshore structures^4^.

The importance of time series data mining has been increasing exponentially in the last decade^5,6^. They are present in different fields of application, e.g. climate^7^, oceanography^8^, biology^9^ and much more. In addition, they are used for different research objectives, such as classification^10^, tipping point detection^11^, forecasting^12^, etc.

Basically, a time series can be defined as temporal data collected in different periods of time. In this sense, the observation of a random variable in regular periods of time can lead to the introduction of noise. That is, if the period between two consecutive observations is much lower than the real cadence of the phenomenon under investigation, a high number of observed values will be very close to the average value of the characteristic studied.

In the context of oceanography and specifically, in the determination of extreme wave height values, if we consider a buoy collecting the wave height value every four hours, then a high proportion of values close to the average wave height will be recorded. This results in the fact that extreme wave heights, which are probably the most interesting ones, will be outnumbered by a set of very similar values without special interest. These non-informative observations have a distorting effect on the measures that could be taken to analyse the variable, because they do not significantly change the mean value but reduce the deviation, increasing the sample size.

Consequently, wave height extreme values will change from being more or less infrequent to atypical or outliers, with the drawbacks that this means for its analysis and prediction. The presence of these extreme values produces a denaturalization of the standard wave height probability distribution. For this reason, it is necessary to define thresholds of wave height from which the extreme wave distributions are considered, where large time series are needed, given that the number of these events every year is very low and depends on the oceanic position of the buoy.

Statistical methods to determine extreme wave heights using the Peaks-Over-Threshold approach (POT) have been significantly improved for several years. Mathiesen et al.^13^ use the POT method along with a Weibull distribution estimated by a maximum likelihood procedure. This is applied to the prediction of individual wave heights associated with high return periods, considering that 100 years or more is enough for the extensive use of ocean’s resources. In 2001, Coles^14^ introduced the GPD-Poisson by fitting a Generalized Pareto Distribution (GPD), which was also used later on^15^.

In 2011, Mazas and Hamm^16^ proposed the determination of extreme wave heights using a POT approach, where a double threshold ($u_1,u_2$) is presented. A low value $u_1$ is set to select both weak and strong storms. Then, a second higher threshold ($u_2$) has to be determined to decide which storms have a statistically extreme behaviour. Tree probability distributions of extreme values are used to determine $u_2$: GPD-Poisson, Weibull and Gamma distributions. To select the best-fitting distribution, two objective criteria based on likelihood (Bayesian Information Criterion^17^, BIC, and Akaike Information Criterion^18^, AIC) are used.

More recently, Petrov et al.^19^ presented a maximum entropy (MaxEnt) method for the prediction of extreme significant wave heights, comparing it with the state of the art methodologies of the Extreme Value Theory (EVT): the GPD and the Generalized Extreme Value distribution (GEV). According to the definition of the MaxEnt principle, the distribution that provides the highest entropy is selected to give more information among all other possible distributions that satisfy the proposed constraints.

As can be seen, all methods are based on selecting a threshold and modelling the distribution of the wave heights over this threshold. Thus, the main problem is how to select this threshold in order to avoid information loss. For that, it could be interesting to model the complete time series with both regular and extreme values and to use this theoretical distribution to fix the threshold for the POT approach. In this paper, we propose a new methodology to determine the distribution of the extreme wave heights considering that the normally distributed extreme wave heights are added as to regular values from a uniform distribution. The reason for choosing a uniform distribution is that, outside a range around the mean, all observations of wave height should be assumed to be part of the problem and never noise. This makes us discard the normal distribution as a contamination distribution. After that, using the estimated theoretical mixed distribution, we set the threshold for the POT methodologies. In this way, we fit several distributions of the values over this threshold and select the best-fitting distribution according to the BIC and AIC criteria.

The novel contributions of this work to applied energy issues are:

- In atmospheric time series, such as wave height^20^, wind power^21^ or fog formation in airports^22,23^, there are many values close to the average. This makes that extreme values of time series, which are the most interesting ones, are hidden by uninteresting values. For this reason, these values have a distorting effect on extreme values. In this paper, we show that regular values do not significantly change the mean value of the time series, but they reduce the deviation by increasing the sample size.
- We propose a new methodology which, up to the author knowledge, has not been applied before to wave height time series. This methodology is able to determine the distribution of the complete time series, taking into account that wave height time series distribution is a mixture of a normal distribution of extreme values and noise from a uniform distribution.
- For adjusting the four parameters needed to define the mixed distribution, we used the method of moments^24^, given that our methodology uses the raw time series.
- When the mixed distribution is estimated, this methodology is used to determine the threshold needed for POT approaches. We assume that using the extreme values situated over a percentile of the theoretical mixed distribution is more reliable than using a predefined value adjusted by a trial and error process. In this way, our methodology is applied to obtain return values for 1, 2, 5, 10, 20, 50 and 100 years for nine real-world wave height time series, using three different percentiles from the mixed distribution.

The rest of paper is organised as follows: section “Methodology” presents the details of the proposed method. Section “Dataset and experimental design” describes the data considered and the characteristics of the experiments, while section “Results and discussion” includes the results and the associated discussion. Finally, section “Conclusion” concludes the paper.

## Methodology

This sections introduces the Extreme Value Theory and presents the proposed methodology of this work.

### Extreme value theory

Extreme Value Theory (EVT) is associated to the maximum sample $M_n = \text {max} (X_1, \ldots , X_n)$, where $(X_1, \ldots , X_n)$ is a set of independent random variables with common distribution function *F*. In this case, the distribution of the maximum observation is given by $Pr(M_n < x) = F^n(x)$. The hypothesis of independence when the *X* variables represent the wave height over a determined threshold is quite acceptable, because, for oceanographic data, it is common to adopt a POT scheme which selects extreme wave height events that are approximately independent^25^. Also, in^26^, authors affirm that “The maximum wave heights in successive sea states can be considered independent, in the sense that the maximum height is dependent only on the sea state parameters and not in the maximum height in adjacent sea states”. This $M_n$ variable is described with one of the three following distributions: Gumbel, Frechet, and Weibull.

One methodology in EVT is to consider wave height time series with the annual maximum approach (AM)^27^, where *X* represents the wave height collected on regular periods of time of one year, and $M_n$ is formed by the maximum values of each year. The statistical behaviour of AM can be described by the distribution of the maximum wave height in terms of Generalized Extreme Value (GEV) distribution:

1 $$\begin{aligned}{}&G(x)= \left\{ \begin{matrix} \exp \left\{ -\left[ 1 + \xi \left( \frac{x - \mu }{\sigma } \right) \right] ^{\frac{1}{\xi }} \right\} , &{} \xi \ne 0,\\ \exp \left\{ -\exp \left( - \left( \frac{x - \mu }{\sigma } \right) \right) \right\} , &{} \xi = 0, \end{matrix} \right. \end{aligned}$$

where:

2 $$\begin{aligned}{}&0< x < 1 + \xi \left( \frac{x-\mu }{\sigma } \right) , \end{aligned}$$

where $-\infty< \mu < \infty$, $\sigma > 0$ and $-\infty< \xi < \infty$. As can be seen, the model has three parameters: location ($\mu$), scale ($\sigma$), and shape ($\xi$).

The estimation of the return values, corresponding to the return period ($T_p$), are obtained by inverting Eq. (1):

3 $$ z_{p}  = \left\{ {\begin{array}{*{20}l}    {\mu  - \frac{\sigma }{\xi }\left[ {1 - \left\{ { - \log (1 - p)} \right\}^{{ - \xi }} } \right],} & {\xi  \ne 0,}  \\    {\mu  - \sigma \log \left\{ { - log(1 - p)} \right\},} & {\xi  = 0,}  \\   \end{array} } \right\}{\text{ }} $$

where $G(z_p) = 1 - p$. Then, $z_p$ will be exceeded once per 1/*p* years, which corresponds to $T_p$.

The alternative method in the EVT context is the Peak-Over-Threshold (POT), where all values over a threshold predefined by the user are selected to be statistically described instead of only the maximum values^28,29^. POT method has become a standard approach for these predictions^13,29,30^. Furthermore, several improvements over the basic approach have been proposed by various authors since then^19,31,32^.

The POT method is based on the fact that if the AM approach uses a GEV distribution (Eq. 1), the peaks over a high threshold should result in the related approximated distribution: the Generalized Pareto Distribution (GPD). The GPD fitted to the tail of the distribution gives the conditional non-exceedance probability $P(X\le x | X > u)$, where *u* is the threshold level. The conditional distribution function can be calculated as:

4 $$ P(X \le x|X{\text{  > }}u) = \left\{ {\begin{array}{*{20}l}    {1 - \left( {1 + \xi ^{*} \left( {\frac{{x - u}}{{\sigma ^{*} }}} \right)} \right)^{{\frac{1}{{\xi ^{*} }}}} ,} & {\xi ^{*}  \ne 0,}  \\    {1 - \exp \left( { - \left( {\frac{{x - u}}{{\sigma ^{*} }}} \right)} \right),} & {\xi ^{*}  = 0.}  \\   \end{array} } \right. $$

There is consistency between the GEV and GPD models, meaning that the parameters can be related to $\xi ^* = \epsilon$ and $\sigma ^* = \sigma + \xi (u - \mu )$. The parameters $\sigma$ and $\xi$ are the scale and shape parameters, respectively. When $\xi \ge 0$, the distribution is referred to as long tailed. When $\xi < 0$, the distribution is referred to as short tailed. The methods used to estimate the parameters of the GPD and the selection of the threshold will be now discussed.

The use of the GPD for modelling the tail of the distribution is also justified by asymptotic arguments in^14^. In this paper, author confirms that it is usually more convenient to interpret extreme value models in terms of return levels, rather than individual parameters. In order to obtain these return levels, the exceedance rates of thresholds have to be determined as $P(X>u)$. In this way, using Eq. (4) ($P(X>x|X>u)=P(X>x)/P(X>u)$) and considering that $z_N$ is exceeded on average every *N* observations, we have:

5 $$\begin{aligned}{}&P(X>u)\left[ 1+\xi ^*\left( \frac{z_N - u}{\sigma ^*}\right) \right] ^{-\frac{1}{\xi ^*}}=\frac{1}{N}. \end{aligned}$$

Then, the *N*-year return level $z_N$ is obtained as:

6 $$\begin{aligned}{}&z_N = u + \frac{\sigma ^*}{\xi ^*}\left[ (N * P(X>u))^{\xi ^ *} - 1 \right] . \end{aligned}$$

There are many techniques proposed for the estimation of the parameters of GEV and GPD. In^19^, authors applied the maximum likelihood methodology (ML) described in^14^. However, the use of this methodology for two parameter distributions (i.e. Weibull or Gamma) has a very important drawback: these distributions are very sensitive to the distance between the high threshold ($u_2$) and the first peak^16^. For this reason, ML could be used with two-parameter distribution when $u_2$ reaches a peak. As this peak is excluded, the first value of the exceedance is as far from $u_2$ as possible. A solution would be to use the three-parameter Weibull and Gamma distributions. However, ML estimation of such distributions is very difficult, and the algorithms usually fit two-parameter distributions inside a discrete range of location parameters^33^.

### Proposed methodology

As stated before, in this paper, we present a new methodology to model this kind of time series considering not only extreme values but also the rest of observations. In this way, instead of selecting the maximum values per a period (usually a year) or defining thresholds in the distribution of these extreme wave heights, which has an appreciable subjective component, we model the distribution of all wave heights, considering that it is a mixture formed by a normal distribution and a uniform distribution. The motivation is that the uniform distribution is associated to regular wave height values which contaminate the normal distribution of extreme values. This theoretical mixed distribution is used then to fix the threshold for the estimation of the POT distributions. Thus, the determination of the threshold will be done in a much more objective and probabilistic way.

Let us consider as a sequence of independent random variables, $(X_1, \ldots , X_n)$ of wave height data. These data follow an unknown continuous distribution. We assume that this distribution is a mixture of two independent distributions: $Y_1 \sim N(\mu , \sigma )$, and $Y_2 \sim U(\mu - \delta , \mu + \delta )$, where $N(\mu , \sigma )$ is a Gaussian distribution, $U(\mu - \delta , \mu + \delta )$ is a uniform distribution, $\mu > 0$ is the common mean of both distributions, $\sigma$ is the standard deviation of $Y_1$, and $\delta$ is the radius of $Y_2$, being $\mu - \delta > 0$. Then $f(x) = \gamma f_1(x) + (1-\gamma ) f_2(x)$, being $\gamma$ the probability that an observation comes from the normal distribution, and *f*(*x*), $f_1(x)$ and $f_2(x)$ are the probability density functions (pdf) of *X*, $Y_1$ and $Y_2$, respectively.

For the estimation of the values of the four above-mentioned parameters ($\mu , \sigma , \delta , \gamma$), the standard statistical theory considers the least squares methods, the method of moments and the maximum likelihood (ML) method. In this context, Mathiesen et al.^13^ found that the least squares methods are sensitive to outliers, although Goda^34^ recommended this method with modified plotting position formulae.

Clauset et al.^35^ show that methods based on least-squares fitting for the estimation of probability-distribution parameters can have many problems, and, usually, the results are biased. These authors propose the method of ML for fitting parametrized models such as power-law distributions to observed data, given that ML provably gives accurate parameter estimates in the limit of large sample size^36^. The ML method is commonly used in multiple applications, e.g. in metocean applications^25^, due to its asymptotic properties of being unbiased and efficient. In this regard, White et al.^37^ conclude that ML estimation outperforms the other fitting methods, as it always yields the lowest variance and bias of the estimator. This is not unexpected, as the ML estimator is asymptotically efficient^37,38^. Also, in Clauset et al.^35^, it is shown, among other properties, that under mild regularity conditions, the ML estimation ${\hat{\alpha }}$ converges almost surely to the true $\alpha$, when considering estimating the scaling parameter ($\alpha$) of a power law in the case of continuous data. It is asymptotically Gaussian, with variance $(\alpha -1)^2/n$. However, the ML estimators do not achieve these asymptotic properties until they are applied to large sample sizes. Hosking and Wallis^39^ showed that the ML estimators are non-optimal for sample sizes up to 500, with higher bias and variance than other estimators, such as moments and probability weighted-moments estimators.

Deluca and Corral^40^ also presented the estimation of a single parameter $\alpha$ associated with a truncated continuous power-law distribution. In order to find the ML estimator of the exponent, they proceed by directly maximizing the log-likelihood $l(\alpha )$. The reason is practical since their procedure is part of a more general method, valid for arbitrary distributions *f*(*x*), for which the derivative of $l(\alpha )$ can be challenging to evaluate. They claim that one needs to be cautious when the value of $\alpha$ is very close to one in the maximization algorithm and replace $l(\alpha )$ by its limit at $\alpha =1$.

Furthermore, the use of ML estimation for two-parameter distributions such as Weibull and Gamma distributions has the drawback^16^ previously discussed. Besides, the ML estimation is known to provide poor results when the maximum is at the limit of the interval of validity of one of the parameters. On the other hand, the estimation of the GPD parameters is subject of ongoing research. A quantitative comparison of recent methods for estimating the parameters was presented by Kang and Song^41^. In our case, having to estimate four parameters, we have decided to use the method of moments, for its analytical simplicity. It is always an estimation method associated with sample and population moments. Besides, adequate estimations are obtained in multi-parametric estimation and with limited samples, as shown in this work.

Considering $\phi$ as the pdf of a standard normal distribution *N*(0, 1), the pdf of $Y_1$ is defined as:

7 $$\begin{aligned}{}&f_1(x) = \frac{1}{\sigma }\phi (z_x), \text { } z_x = \frac{x - \mu }{\sigma }, \text { } x \in {\mathbb {R}}. \end{aligned}$$

The pdf of $Y_2$ is:

8 $$\begin{aligned}{}&f_2(x) = \frac{1}{2\delta }, \text { } x \in (\mu -\delta ,\mu +\delta ). \end{aligned}$$

Consequently, the pdf of *X* is:

9 $$\begin{aligned}{}&f(x) = \gamma f_1(x) + (1 - \gamma ) f_2(x), \text { } x \in {\mathbb {R}}. \end{aligned}$$

To infer the values of the four parameters of the wave height time series ($\mu$, $\sigma$, $\delta$, $\gamma$), we define, for any symmetric random variable with respect to the mean $\mu$ with pdf *g* and finite moments, a set of functions in the form:

10 $$\begin{aligned}{}&U_k(x) = \int _{|t - \mu | \ge x}|t - \mu |^k g(t)dt, \text { } x \ge 0, \text { } k = 1,2,3, \ldots , \end{aligned}$$

or because of its symmetry:

11 $$\begin{aligned}{}&U_k(x) = 2\int _{x+\mu }^{\infty }(t - \mu )^k g(t)dt, \text { } k = 1,2,3, \ldots . \end{aligned}$$

These functions are well defined for the same moments of the variable *x*, because:

12 $$\begin{aligned}{}&U_k(x)< \int _{-\infty }^{\infty }|t - \mu |^k g(t)dt < \infty , \text { } k = 1,2,3, \ldots . \end{aligned}$$

Particularly, for the normal and uniform distributions, all the moments are finite, and the same happens for all the $U_k(x)$ functions. This function measures, for each pair of values *x* and *k*, the bilateral tail from the value *x* of the moment with respect to the mean of order *k* of the variable. It is, therefore, a generalization of the concept of probability tail, which is obtained for $k = 0$.

Now, if we denote the corresponding moments for the distributions $Y_1$ and $Y_2$ by $U_{k,1}(x)$ and $U_{k,2}(x)$, it is verified that:

13 $$\begin{aligned}{}&U_k(x) = \gamma U_{k,1}(x) + (1 - \gamma ) U_{k,2}(x). \end{aligned}$$

Then, to calculate the function $U_k(x)$, we just need to calculate the functions $U_{k,1}(x)$ and $U_{k,2}(x)$.

#### Calculation $U_k$ for the uniform distribution ($U_{k,2}$)

From the definition of $f_2(x)$ and $U_k(x)$, if $\mu > \delta$:

14 $$\begin{aligned}{}&U_{k,2}(x) = 2 \int _{\mu +x}^{\mu +\delta }(t-\mu )^k\frac{1}{2\delta }dt = \left. \frac{(t-\mu )^{k+1}}{(k+1)\delta } \right| _{\mu +x}^{\mu +\delta } = \frac{\delta ^{k+1} - x^{k+1}}{(k+1)\delta }, \end{aligned}$$

then,

15 $$\begin{aligned} & U_{k,2}(x) =\left\{ \begin{array}{ll} \frac{\delta ^{k+1} - x^{k+1}}{(k+1)\delta } & 0 \le x \le \delta ,\\ \quad \quad 0 & \quad x >\delta . \end{array}\right. \end{aligned}$$

#### Calculation $U_k$ for the normal distribution ($U_{k,1}$)

From the definition of the $f_1(x)$ and $U_k(x)$, we have:

16 $$\begin{aligned}{}&U_{k,1}(x)=\frac{2}{\sigma }\int _{\mu +x}^\infty (t-\mu )^k\phi \left( \frac{t-\mu }{\sigma } \right) dt. \end{aligned}$$

Let the variable *u* be in the form $u = \frac{t-\mu }{\sigma }$, then:

17 $$\begin{aligned}{}&U_{k,1}(x)=2\int _{\frac{x}{\sigma }}^\infty (u\sigma )^k\phi (u)du = \sigma ^k\Upsilon _k\left( \frac{x}{\sigma } \right) , \end{aligned}$$

where $\Upsilon _k = 2 \int _{x}^\infty (u)^k\phi (u)du$. $\Upsilon _k(z)$ is the $U_k$ function calculated for a *N*(0, 1) distribution, which will be then updated with values of $k=1,2,3$.

##### Proposition I

The following equations are verified:

18 $$\begin{aligned}{}&\Upsilon _1(x) = 2 \int _{x}^\infty u\phi (u)du = 2\phi (x),\end{aligned}$$

19 $$\begin{aligned}{}&\Upsilon _2(x) = 2 \int _{x}^\infty u^2\phi (u)du = 2(1-\Phi (x)+x\phi (x)),\end{aligned}$$

20 $$\begin{aligned}{}&\Upsilon _3(x) = 2 \int _{x}^\infty u^3\phi (u)du = 2(2+x^2)\phi (x), \end{aligned}$$

where $\Phi$ is the cumulative distribution function (CDF) of the *N*(0, 1) distribution. The demonstration is included below.

The three equations can be obtained using integration by parts, but it is easier to derive the functions $\Upsilon _k(x)$ to check the result. For the definition of the functions, for each value of *k*, we have:

21 $$\begin{aligned}{}&\Upsilon _k^{'}(x) = \frac{\partial \Upsilon _k(x)}{\partial x} = -2x^k\phi (x). \end{aligned}$$

Taking into account that $\frac{\partial \phi (x)}{\partial x}=-x\phi (x)$, and $\frac{\partial \Phi (x)}{\partial x}=\phi (x)$:

22 $$\begin{aligned} \frac{\partial 2\phi (x)}{\partial x}&= -2x\phi (x)= \Upsilon _1^{'}(x), \end{aligned}$$

23 $$\begin{aligned} \frac{\partial (2(1-\Phi (x)+x\phi (x)))}{\partial x}&= 2(-\phi (x)+\phi (x)-x^2\phi (x)) = \nonumber \\&=-2x^2\phi (x)= \Upsilon _2^{'}(x), \end{aligned}$$

24 $$\begin{aligned} \frac{\partial (2(2+x^2)\phi (x))}{\partial (x)}&=2(2x\phi (x)-(2+x^2)x\phi (x))=\nonumber \\&=-2x^3\phi (x)=\Upsilon _3^{'}(x). \end{aligned}$$

Therefore, the left and right sides of the previous equations differ in, at most, a constant. To verify that they are the same, we check the value $x=0$:

25 $$\begin{aligned}{}&\Upsilon _1(0) = 2 \int _0^\infty u \phi (u)du = \sqrt{\frac{2}{\pi }},\end{aligned}$$

26 $$\begin{aligned}{}&\Upsilon _2(0) = 2 \int _0^\infty u^2 \phi (u)du = 1,\end{aligned}$$

27 $$\begin{aligned}{}&\Upsilon _3(0) = 2 \int _0^\infty u^3 \phi (u)du = 2 \sqrt{\frac{2}{\pi }}, \end{aligned}$$

which match with the right sides of Eqs. (18)–(20):

28 $$\begin{aligned}{}&\Upsilon _1(0) = 2 \phi (0) = \sqrt{\frac{2}{\pi }},\end{aligned}$$

29 $$\begin{aligned}{}&\Upsilon _2(0) = 2 (1-\Phi (0)) = 1,\end{aligned}$$

30 $$\begin{aligned}{}&\Upsilon _3(0) = 2 (2)\phi (0) = 2 \sqrt{\frac{2}{\pi }}. \end{aligned}$$

Substituting these results in Eq. (17) we have:

31 $$\begin{aligned}{}&U_{1,1}=\sigma \Upsilon _1 \left( \frac{x}{\sigma }\right) = 2 \sigma \phi \left( \frac{x}{\sigma }\right) ,\end{aligned}$$

32 $$\begin{aligned}{}&U_{2,1}=\sigma ^2 \Upsilon _2 \left( \frac{x}{\sigma }\right) = 2\sigma ^2 \left( 1 - \Phi \left( \frac{x}{\sigma } \right) + \frac{x}{\sigma }\phi \left( \frac{x}{\sigma } \right) \right) ,\end{aligned}$$

33 $$\begin{aligned}{}&U_{3,1}=\sigma ^3 \Upsilon _3 \left( \frac{x}{\sigma }\right) = 2 \sigma ^3\left( 2 + \left( \frac{x}{\sigma } \right) ^2 \right) \phi \left( \frac{x}{\sigma }\right) . \end{aligned}$$

These functions will be the base to estimate the parameters of the distribution of variable *X*, except in the case of $\mu$, as we will comment later. The estimates will be made with the corresponding $U_k$ sample estimates, defined in the following Section.

#### Sample estimates of $U_k$

For each value of *k* and $x \ge 0$, the sample estimator of $U_k$ obtained by the method of moments is:

34 $$\begin{aligned}{}&u_k(x)=\frac{1}{n} \sum _{|x_i-\mu |\ge x}|x_i - \mu |^k, \end{aligned}$$

which has the properties described in the following propositions.

##### Proposition II

 The estimator $u_k(x)$ is an unbiased estimator of $U_k(x)$. For the demonstration, we first rewrite $u_k$ in the form:

35 $$\begin{aligned}{}&u_k(x)=\frac{1}{n} \sum _{i=1}^n|x_i - \mu |^kI\{|x_i - \mu |\ge x\}, \end{aligned}$$

where *I* is the indicator function. Considering the previous expression, we check the condition of an unbiased estimator:

36 $$\begin{aligned} E(u_k(x))&= \frac{1}{n} \sum _{i=1}^n E(|x_i - \mu |^kI\{|t - \mu |\ge x\}) = \nonumber \\&=E(|t - \mu |^kI\{|t - \mu |\ge x\} = \nonumber \\&= \int _{|t - \mu |\ge x}|t-\mu |^kg(t)dt = U_k(x). \end{aligned}$$

##### Proposition III

The estimator $u_k(x)$ is a consistent estimator of $U_k(x)$. Considering again Eq. (35) for the variance of $u_k(x)$ we have:

37 $$\begin{aligned}{}&V(u_k(x)) = \nonumber \\&\quad =\frac{1}{n^2} \sum _{i=1}^n V(|x_i - \mu |^kI\{|t - \mu |\ge x\}) = \frac{1}{n}V(|t - \mu |^kI\{|t - \mu |\ge x\}) = \nonumber \\&\quad =\frac{1}{n}\left( E (|t - \mu |^{2k}I\{|t - \mu |\ge x\}) - E^2(|t - \mu |^kI\{|t - \mu |\ge x\})\right) =\nonumber \\&\quad =\frac{1}{n}(U_{2k}(x)-U_{k}^2(x))\overset{n\rightarrow \infty }{\rightarrow }0, \end{aligned}$$

taking into account that $I^2\{.\} = I\{.\}$.

#### Parameter estimation of the mixed distribution of *X*

The estimates are based on the $u_k(0)$ values, for $k=1,2,3$, which estimate the corresponding population parameters.

Estimation of $\mu$ Given that the mean value of both distributions (uniform and normal) is the same, this value is not affected by the mixture. Therefore, the natural estimator is

38 $$\begin{aligned}{}&{\hat{\mu }} = {\bar{x}} = \frac{1}{n} \sum _{i=1}^n x_i.&\end{aligned}$$

#### Estimation of $\sigma$, $\delta$, and $\gamma$ parameters

Applying the method of moments, we have the following three-equation system:

39 $$\begin{aligned}{}&U_k(0)=u_k(0) \text {, } k=1,2,3.&\end{aligned}$$

The reason for choosing the origin is that it has the maximum amount of information about the $u_k(x)$ functions defined in Eq. (34). If a nonzero *x* value is chosen, the estimate will discard all observations in the interval $(\mu - x, \mu + x)$. Substituting Eqs. (15), (31), (32) and (33) in Eq. (13), the resulting equation system is:

40 $$\begin{aligned}{}&\gamma U_{1,1}(0) + (1-\gamma )U_{1,2}(0)=\gamma \sigma \sqrt{\frac{2}{\pi }}+(1-\gamma )\frac{\delta }{2}=u_1(0),\end{aligned}$$

41 $$\begin{aligned}{}&\gamma U_{2,1}(0) + (1-\gamma )U_{2,2}(0) = \gamma \sigma ^2 + (1-\gamma )\frac{\delta ^2}{3} = u_2(0),\end{aligned}$$

42 $$\begin{aligned}{}&\gamma U_{3,1}(0) + (1-\gamma )U_{3,2}(0)=\gamma \sigma ^3 2\sqrt{\frac{2}{\pi }}+(1-\gamma )\frac{\delta ^3}{4}=u_3(0), \end{aligned}$$

where the solution must satisfy: ${\hat{\sigma }},{\hat{\delta }} > 0$ and $\gamma \in [0,1]$.

#### Adjustment to the mixed distribution

To contrast if the obtained estimators are valid, we could see if the set of observations $\{x_1,\ldots ,x_n\}$ fit the pdf of the final distribution:

43 $$\begin{aligned}{}&{\hat{f}}(x) = {\hat{\gamma }} \hat{f_1}(x)+ (1-{\hat{\gamma }})\hat{f_2}(x) \text {, } x \in {\mathbb {R}},&\end{aligned}$$

where:

44 $$\begin{aligned}{}&\hat{f_1}(x) = \frac{1}{{\hat{\sigma }}}\phi \left( \frac{x - {\hat{\mu }}}{{\hat{\sigma }}}\right) \text {, } x \in {\mathbb {R}},&\end{aligned}$$

and:

45 $$\begin{aligned}{}&\hat{f_2}(x)=\frac{1}{2{\hat{\delta }}} \text {, } x \in ({\hat{\mu }} - {\hat{\delta }}, {\hat{\mu }} + {\hat{\delta }}).&\end{aligned}$$

For this purpose, a test that can be used is the Kolmogorov-Smirnov test. The one-sample Kolmogorov-Smirnov test^42^ is commonly used to examine whether samples come from a specific distribution function by comparing the observed cumulative distribution function with an assumed theoretical distribution. The Kolmogorov-Smirnov statistic *Z* is computed from the largest difference (in absolute value) between the observed and theoretical cumulative distribution. In this way, *Z* is the greatest vertical distance between empirical distribution function *S*(*x*) and the specified hypothesized distribution function $F^*(x)$, which can be calculated as:

46 $$\begin{aligned}{}&Z=\max _x|F^*(x) - S(x)|,&\end{aligned}$$

where the null hypothesis is $H_0: F(x) = F^*(x)$ for all $-\infty< x < \infty$, and the alternative hypothesis is $H_1: F(x) \ne F^*(x)$ for at least one value of *x*, *F*(*x*) being the true distribution. If *Z* exceeds the $1\text {-}\alpha$ quantile value ($Q(1-\alpha )$), then we reject $H_0$ at the level of significance of $\alpha$. When the number of observations *n* is large, the $Q(1-\alpha )$ value can be approximated as^43^:

47 $$\begin{aligned}{}&Q(1-\alpha ) = \frac{\sqrt{-0.5 \log {(\frac{\alpha }{2})}}}{\sqrt{n}}.&\end{aligned}$$

### Using the theoretical mixed distribution to fix the threshold of the POT approaches

In this paper, when the mixed distribution is estimated, we use it to set the threshold for estimating the POT distributions. We assume that using the points which are situated over a percentile of the theoretical mixed distribution is more reliable than using a threshold value predefined by a trial and error procedures. Identifying extreme values when studying a phenomenon is supported by the determination of a limit value or a probability threshold. Since the consideration of extreme is determined by an unusual deviation from the central values of the distribution of the phenomenon under investigation, we understand that the probabilistic approach is preferred. In our work, we consider the 95%, 97.5% and 99% percentiles as possible thresholds.

In this way, a new sample of independent random variables is defined by $Z = (z_1, z_2, \ldots , z_M)$, where $Z = X > u$, *u* being the threshold and *M* being the number of exceedances. In this work, three distributions are fitted for the threshold exceedance distribution:

- The first one is the GPD^44^, whose cumulative function is defined in Eq. (4).
- The second distribution is the Gamma distribution, with the following cumulative function:
  48 $$\begin{aligned} F(z;\xi ,\sigma ) = \frac{\gamma (\xi ,\frac{z}{\sigma })}{\Gamma (\xi )}, \end{aligned}$$
  where $\gamma$ is the lower incomplete gamma function, and $\Gamma$ is the *Gamma* function.
- Finally, the Weibull distribution is also considered:
  49 $$\begin{aligned} F(z;\xi ,\sigma ) = 1 - \exp \left[ -\left( \frac{z}{\sigma } \right) ^\xi \right] . \end{aligned}$$

These three distributions are adjusted to the exceedances using the Maximum Likelihood Estimator (MLE)^13^. After that, we select the best fit based on two objective criteria: BIC^17^ and AIC^18^. On the one hand, BIC minimizes the bias between the fitted model and the unknown true model:

50 $$\begin{aligned} \text {BIC} = -2\ln L + k_p \ln M, \end{aligned}$$

where *L* is the likelihood of the fit, *M* is the sample size (in our case, the number of exceedances) and $k_p$ the number of parameters of the distribution. On the other hand, AIC gives the model providing the best compromise between bias and variance:

51 $$\begin{aligned} \text {AIC} = -2\ln L + 2 k_p. \end{aligned}$$

Both criteria need to be minimized.

When the best-fitted distribution is obtained, the return period T ($Hs_T$) is calculated, and then the confidence intervals are computed. As can be seen in the experimental section, the GPD is the best distribution for all cases. The quantile for the GPD is:

52 $$\begin{aligned} Hs_T = \mu + \frac{\sigma }{\xi }\left[ 1 - (\lambda T)^{-\xi } \right] , \end{aligned}$$

where $\lambda$ is the number of exceedances per year.

Finally, confidence intervals are also computed. For that, many authors use the classical asymptotic method^14^. However, Mathiesen et al. advocate the use of Monte-Carlo (MC) simulation techniques. Also, Mackay and Johanning^26^ proposed a storm-based MC method for calculating return periods of individual wave and crest heights. In the MC method, a random realisation of the maximum wave height in each sea state is simulated from the metocean parameter time series, and the GPD is fitted to storm peak wave heights exceeding some threshold. Mackay and Johanning^26^ showed that using $n=1000$ is sufficient to obtain a stable estimation, although in our case, we have considered $n=100000$ following the work of^16^. In^16^, as in our work, authors used the MC simulation method, and, after 100000 iterations, the $90\%$ confidence interval is obtained using the percentiles [$Hs_{T,5\%};Hs_{T,95\%}$] of the 100000 $Hs_T$ values obtained with the procedure.

## Dataset and experimental design

### Dataset

As stated before, the objective of this work is to model wave height time series where extreme values are present. For this reason, we evaluate the performance of the proposed methodology in several real-world wave height time series from different locations:

- Gulf of Alaska: two wave height time series collected from the National Data Buoy Center of the USA^45^ in the Gulf of Alaska have been used. The buoys have the registration numbers 46001 and 46075. For the two buoys, one value every six hours is considered. The buoy 46001 is an offshore buoy placed in the coordinates 56.23N 147.95W, and data from 1st January 2008 to 31st December 2013 is considered, with a total of 8767 observations. On the other hand, 46075 is an offshore buoy whose coordinates are 53.98N 160.82W and data from 1st January 2011 to 31st December 2015 are collected in this buoy (7303 observations).
- Puerto Rico: a total of six offshore buoys from Puerto Rico have been selected in our experiments to evaluate the proposed methodology. These buoys also belong to the NDBC of the USA, with registration ids 41043, 41044, 41046, 41047, 41048 and 41049. One value every six hours is considered, and data from 1st January 2011 to 31st December 2015 are used (7303 observations for each one). The geographical coordinates for each buoy are 21.13N 64.86W, 21.58N 58.63W, 23.83N 68.42W, 27.52N 71.53W, 31.86N 69.59W, and 27.54N 62.95W, respectively.
- Spain: this dataset comes from the SIMAR-44 hindcast database provided by Puertos del Estado (Spain). The point is placed in the Strait of Gibraltar, whose coordinates are 36N 6W. One value every three hours is considered in this dataset from 1st January 1959 to 31 December 2000, forming a set of 122278 observations. Note that, it is the largest time series in our experiments. Given that the time series includes 42 years, we can estimate long return periods of wave height.

**Figure 1:**
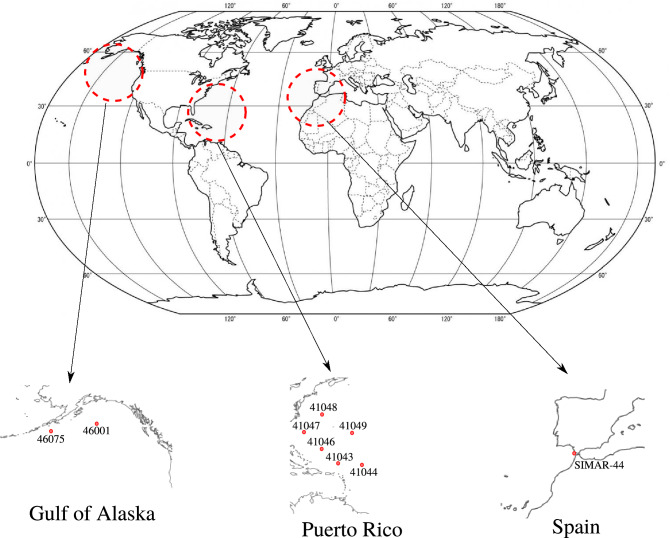
Locations of the different buoys considered for the experimentation.

**Figure 2:**
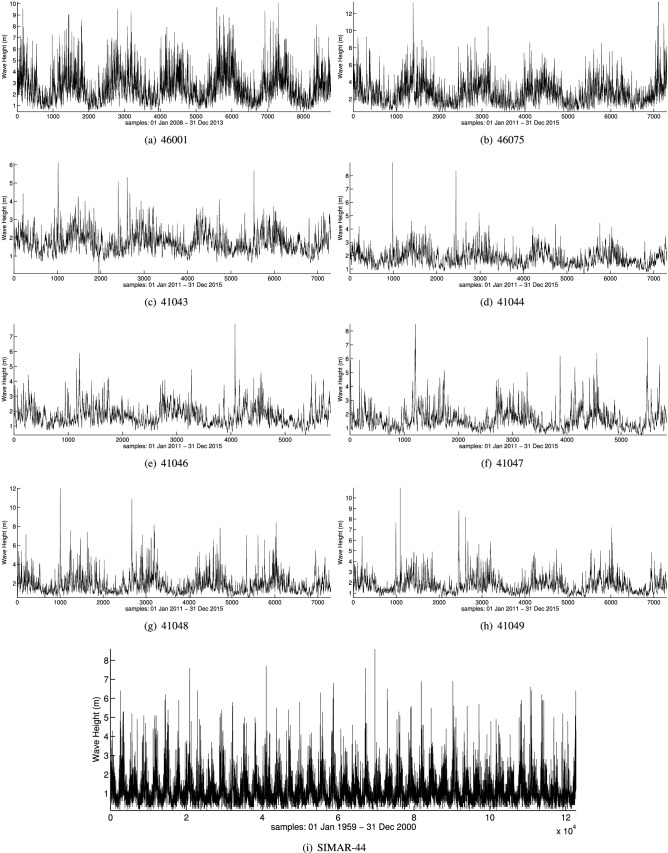
Graphical representation of the time series recorded for every buoy.

The summary of the information for each time series can be seen in Table 1 which includes the type of buoy, the location, the geographical coordinates, the number of observations, the mean values of the time series (Hs), and the maximum values of each one. The map location can be observed in Fig. 1, while the representation of the time series are shown in Fig. 2.

**Table 1:** Characteristics of the time series recorded for every buoy.

Id	Type	Location	Coordinates	# Observations	Average Hs (m)	Max Hs (m)
46001	Offshore	Alaska	56.23N 147.95W	8767	2.65	10.17
46075	Offshore	Alaska	53.98N 160.82W	7303	2.72	13.39
41043	Offshore	Puerto Rico	21.13N 64.86W	7303	1.76	6.12
41044	Offshore	Puerto Rico	21.58N 58.63W	7303	1.84	8.98
41046	Offshore	Puerto Rico	23.83N 68.42W	7303	1.71	7.85
41047	Offshore	Puerto Rico	27.52N 71.53W	7303	1.63	8.51
41048	Offshore	Puerto Rico	31.86N 69.59W	7303	1.85	12.07
41049	Offshore	Puerto Rico	27.54N 62.95W	7303	1.78	10.96
SIMAR-44	Coastal	Spain	36.00N 6.00W	122278	1.09	8.60

### Experimental design

**Figure 3:**
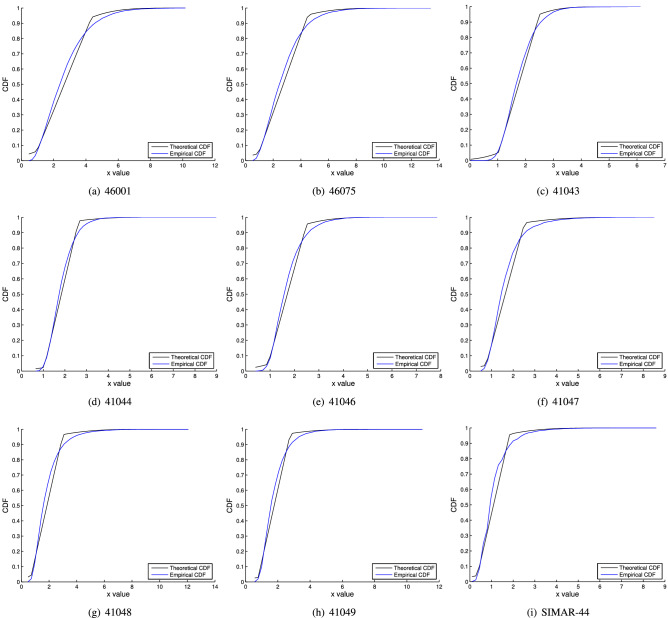
Estimated theoretical distribution versus empirical distribution in all wave height time series considered.

The experimental design for the time series under study is presented in this subsection. We divide the experiments in three stages:

- Firstly, a Kolmogorov-Smirnov test is applied to determine whether the wave height distributions follow a normal distribution. That is, their distributions fit a simple Gaussian. The reason behind applying this test is that, if the wave height distributions follow a normal distribution, using the proposed methodology will not make sense. If this is not the case, we will proceed with the following points.
- Secondly, the methodology is tested on the raw time series presented in the previous subsection. The algorithm estimates the parameters of the mixed distribution $(\mu , \sigma , \delta , \gamma )$ for each wave height time series, and then, the Kolmogorov-Smirnov test is applied to check if the estimated distribution corresponds to the empirical distribution of the data. It is important to mention that the Kolmogorov-Smirnov test is applied considering $n=50$, which is an acceptable value for the Eq. (47), that is, we calculate the CDF of the estimated theoretical function and the empirical one in 50 intervals. Graphically, in this paper, we show the comparison between the theoretical distribution (estimated) and the empirical one (Fig. 3).
- Finally, as we stated in previous sections, we use the theoretical mixed distribution to establish the threshold. In this sense, we delete the values below the threshold, and we fit the GPD, Gamma and Weibull distributions with the remaining values (those which are higher than the threshold). Based on two objective criteria, BIC and AIC, we select the best-fitted distribution and, finally, the return values of this distribution for the following return periods in years $T=(1,2,5,10,20,50,100)$ are calculated.

## Results and discussion

As mentioned above, the first phase of the experimentation is to check that the distributions of the wave height time series do not follow a normal distribution. The Kolmogorov-Smirnov test obtains Z values between 0.6 and 0.8, while the critical values are around 0.016. Moreover, the *p*-value is 0 in all cases and, therefore, lower than any $\alpha$ value. Thus, for all time series, the null hypothesis is rejected, and it can be stated that the wave height time series distribution does not fit a simple Gaussian. We, therefore, proceed to part two of the experimentation.

For the mixed distribution proposed in this paper, the estimates and the Kolmogorov-Smirnov test results are shown in Table 2. As can be seen, the estimation of the $\mu$ parameter is the same than the mean value of the time series (see Table 1), because we have used the sample mean as estimator (see section “Proposed methodology”). $\sigma$ estimation seems to be very high with respect to the mean. It makes sense given that the estimation is made with approximately 7000 points, the variance needing to be high. $\delta$ has values in the interval (0.74,1.80) because there is wave height data that, although not very small, contaminates the normal distribution (in intervals of three months, the parameter value is lower). $\gamma$, which is the probability that an observation comes from the normal distribution, is very low. Again, this makes sense because of the high amount of data which are not extreme values and represent regular waves (uniform distribution). The Kolmogorov-Smirnov test does not reject the null hypothesis for all cases, $Z < Q(1-\alpha )$, confirming that the estimated parameters of the mixed distribution correspond to the empirical values. For this reason, we can accept the theory proposed in this paper as a good method to estimate the theoretical distribution in wave height time series. Note that the *Z* values are lower in those time series whose mean value is higher, so the wave height time series collected from buoys 46001 and 46075 are better adjusted with this distribution, while the Spanish time series results in a worse fit. The results of the Kolmogorov-Smirnov test can be complementary analysed with the representation of the empirical and theoretical distribution, as can be observed in Fig. 3. The graphs show how the estimated theoretical distributions are adapted to the empirical distributions in each database.

**Table 2:** Parameter estimation and Kolmogorov-Smirnov test results.

Id	${\hat{\mu }}$	${\hat{\sigma }}$	${\hat{\delta }}$	${\hat{\gamma }}$	*Z*	$Q(1-\alpha )$
46001	2.652597	2.082763	1.708683	0.296738	0.081194	0.192065
46075	2.724890	2.522156	1.799095	0.189406	0.080575	0.192065
41043	1.762838	0.956801	0.743943	0.224906	0.086916	0.192065
41044	1.836434	1.449356	0.795858	0.077810	0.107365	0.192065
41046	1.705895	1.236797	0.793447	0.170138	0.099714	0.192065
41047	1.633332	1.853012	0.893645	0.113544	0.110250	0.192065
41048	1.849044	2.435167	1.158171	0.109262	0.119285	0.192065
41049	1.777286	2.023050	0.998251	0.091232	0.132657	0.192065
SIMAR-44	1.093372	1.580551	0.748225	0.125561	0.142356	0.192065

For the third experiment, Table 3 shows the values of the BIC and AIC criteria when the GPD, Gamma and Weibull distribution are fitted using the values over the threshold determined by the percentiles 95%, 97.5% and 99% of the theoretical mixed distribution. The number of POTs (*M*) and the number of peaks per year ($\lambda$) are also included. As can be seen, the higher the percentile, the lesser number of peaks per year, because the number of POTs will be much lower. The results confirm that the best fitted distribution for all databases and for all percentiles is the GPD.

There exist a perfect correlation between the values of BIC and AIC for the three percentiles (0.977, 0.998 and 1.000, respectively), for the three distributions and the nine time series. In Table 3, it can be seen that the number of annual peaks is more reasonable when considering the 97.5% and 99% percentiles. This is because the lower the threshold, the more the number of waves from the uniform distribution, i.e. non-extreme waves, are contaminating the distribution of extreme waves, the more the number of less relevant peaks. For instance, in buoy 46001, the BIC value for the GPD is 786.42 and 313.09 respectively, a 21.57% and 19.61% lower than the value for the Gamma distribution, and a 31.39% and 29.05% lower than the value for the Weibull distribution. These results differ from those obtained by^16^ for the SIMAR-44 time series, where GPD gives poor results with respect to these criteria when compared to Gamma; but it is important to mention that we use a 3-parameter GPD instead of a 2-parameter one.

**Table 3:** BIC and AIC criterion for the estimated distributions of the POT method.

Id		Percentile 95%				Percentile 97.5%				Percentile 99%			
		*M*	$\lambda$	BIC	AIC	*M*	$\lambda$	BIC	AIC	*M*	$\lambda$	BIC	AIC
46001	GPD	806	134.33	662.72	653.33	379	63.17	786.42	774.61	154	25.67	313.09	303.98
	Gamma			2193.33	2183.95			1002.74	994.87			389.46	383.38
	Weibull			2497.93	2488.54			1146.18	1138.30			441.27	435.20
46075	GPD	786	157.20	1894.56	1880.56	337	67.40	818.69	807.23	126	25.20	290.39	281.88
	Gamma			2381.82	2372.49			1025.61	1017.97			389.93	384.26
	Weibull			2719.86	2710.53			1188.91	1181.27			458.29	452.62
41043	GPD	784	156.80	302.62	288.63	298	59.60	79.40	68.20	94	18.80	49.64	41.98
	Gamma			820.51	811.18			375.38	367.92			158.16	153.06
	Weibull			1307.63	1298.30			574.64	567.17			207.79	202.69
41044	GPD	758	151.60	346.78	332.89	694	138.80	320.77	307.14	110	22.00	50.51	42.41
	Gamma			1018.04	1008.78			947.71	938.63			249.05	243.65
	Weibull			1638.67	1629.41			1521.01	1511.93			328.35	322.95
41046	GPD	669	167.25	606.02	592.50	280	70.00	238.24	227.33	92	23.00	62.41	54.84
	Gamma			1040.63	1031.62			449.81	442.54			173.41	168.36
	Weibull			1399.50	1390.49			628.37	621.10			235.26	230.21
41047	GPD	629	157.25	1064.67	1051.34	316	79.00	580.17	568.91	97	24.25	185.58	177.85
	Gamma			1503.31	1494.42			775.16	767.65			253.51	248.36
	Weibull			1749.18	1740.29			910.31	902.80			295.82	290.67
41048	GPD	806	161.20	1776.19	1762.11	412	82.40	971.75	959.69	120	24.00	301.70	293.34
	Gamma			2320.91	2311.53			1231.09	1223.04			368.35	362.77
	Weibull			2626.43	2617.05			1392.24	1384.20			421.23	415.66
41049	GPD	811	162.20	1227.71	1213.61	558	111.60	895.71	882.74	112	22.40	226.25	218.09
	Gamma			1870.43	1861.03			1337.14	1328.49			324.23	318.80
	Weibull			2277.40	2268.00			1624.41	1615.76			378.43	372.99
SIMAR-44	GPD	13847	329.69	16998.99	16976.38	5768	137.33	8345.27	8325.29	1908	45.43	2867.27	2850.61
	Gamma			28375.75	28360.68			12646.92	12633.60			4089.08	4077.97
	Weibull			33396.55	33381.48			14701.35	14688.03			4842.63	4831.52

Finally, the return values and the confidence intervals for each dataset considering the different thresholds are summarized in Table 4. We have considered return periods of $T\in \{1,2,5,10,20,50,100\}$ years. If we compare the obtained return values and the confidence intervals with respect to the ones obtained by Mazas and Hamm^16^, for SIMAR-44 time series, we can see that the results are not the same due to the differences in the thresholds, and because they consider 44 years instead of 42, as the first and the last year are used although they are not complete. We agree with the authors in that work in the sense that choosing the right threshold is not always a straightforward issue. For example, if we consider the percentile 97.5% of the theoretical mixed distribution, the return values and the confidence intervals are quite similar to the ones obtained by Mazas (with the slight differences commented above). With respect to the values obtained for the rest of the buoys, up to our knowledge, there are not other reference values. These estimations are approximate, given the reduced length of the time series (six years for buoy 46001 and five for the other buoys). If we compare them with the extreme values that appear in Table 1, we can see that, for the buoys 46075, 41043, 41046, the confidence intervals for the 95% percentile tend to contain these values more frequently, for the buoys 41047, 41048, 41049 and SIMAR-44, the confidence intervals are more adjusted, and, for the buoys 46001 and 41044, there are no confidence intervals that contain them.

**Table 4:** Return values and confidence intervals for the GPD distribution considering $T=(1,2,5,10,20,50,100)$ and the percentiles $95\%$, $97.5\%$, and $99\%$.

Id	T	Percentile 95%		Percentile 97.5%		Percentile 99%	
		$Hs_T$	Confidence Interval	$Hs_T$	Confidence Interval	$Hs_T$	Confidence Interval
46001	100	23.50	18.25–32.75	20.65	15.17–32.21	28.71	18.17–62.30
	50	21.46	17.00–29.06	18.95	14.46–28.29	25.09	16.99–50.77
	20	18.97	15.47–24.49	16.87	13.31–23.42	21.01	15.12–37.18
	10	17.22	14.34–21.59	15.40	12.56–20.75	18.38	13.84–29.61
	5	15.60	13.18–19.17	14.03	11.70–18.04	16.09	12.60–24.10
	2	13.61	11.89–16.11	12.35	10.66–15.08	13.51	11.28–18.14
	1	12.22	10.88–14.15	11.18	9.93–13.15	11.84	10.32–14.79
46075	100	16.24	12.99–21.77	16.69	12.48–25.15	12.59	9.79–21.29
	50	15.39	12.49–19.95	15.78	12.11–22.95	12.22	9.67–19.40
	20	14.28	11.85–18.21	14.59	11.59–20.30	11.70	9.48–17.23
	10	13.44	11.34–16.68	13.70	11.15–18.38	11.27	9.40–15.90
	5	12.60	10.78–15.27	12.81	10.69–16.51	10.82	9.19–14.24
	2	11.49	10.06–13.58	11.64	10.00–14.26	10.18	8.94–12.58
	1	10.64	9.49–12.23	10.77	9.50–12.82	10.18	8.94–12.58
41043	100	6.47	5.38–8.34	4.68	4.04–5.93	4.58	3.99–6.48
	50	6.20	5.26–7.81	4.61	4.02–5.72	4.54	3.97–6.23
	20	5.85	5.02–7.10	4.50	3.97–5.46	4.48	3.96–5.90
	10	5.57	4.84–6.63	4.41	3.94–5.23	4.42	3.94–5.59
	5	5.29	4.66–6.21	4.30	3.89–5.03	4.35	3.93–5.33
	2	4.93	4.43–5.62	4.15	3.81–4.69	4.23	3.88–4.96
	1	4.64	4.24–5.21	4.02	3.73–4.46	4.13	3.84–4.66
41044	100	5.10	4.42–6.19	5.06	4.40–6.15	4.03	3.78–4.65
	50	4.99	4.36–5.96	4.95	4.32–5.94	4.02	3.78–4.61
	20	4.83	4.28–5.66	4.80	4.26–5.67	4.01	3.78–4.54
	10	4.70	4.21–5.45	4.68	4.18–5.43	4.00	3.78–4.49
	5	4.56	4.12–5.19	4.55	4.11–5.21	3.98	3.77–4.42
	2	4.36	4.00–4.87	4.35	3.99–4.89	3.95	3.76–4.30
	1	4.20	3.89–4.62	4.19	3.88–4.62	3.91	3.75–4.21
41046	100	7.53	6.01–10.21	6.50	5.13–9.55	4.87	4.26–6.83
	50	7.20	5.86–9.49	6.29	5.07–8.94	4.83	4.25–6.60
	20	6.75	5.62–8.63	6.00	4.96–8.11	4.77	4.24–6.22
	10	6.41	5.43–7.99	5.77	4.83–7.49	4.72	4.22–5.98
	5	6.06	5.21–7.35	5.53	4.72–6.96	4.65	4.20–5.70
	2	5.60	4.92–6.59	5.19	4.55–6.27	4.55	4.17–5.31
	1	5.24	4.68–6.04	4.92	4.41–5.76	4.45	4.14–5.05
41047	100	7.83	6.25–10.50	10.37	7.58–16.37	9.35	6.55–19.55
	50	7.57	6.14–9.99	9.85	7.41–15.03	8.98	6.45–17.26
	20	7.19	5.95–9.22	9.15	7.06–13.36	8.47	6.34–14.93
	10	6.89	5.78–8.63	8.61	6.82–11.91	8.06	6.21–13.08
	5	6.58	5.58–8.03	8.06	6.54–10.81	7.63	6.09–11.60
	2	6.13	5.32–7.33	7.33	6.15–9.27	7.04	5.85–9.66
	1	5.78	5.09–6.76	6.77	5.81–8.32	6.57	5.65 -8.38
41048	100	10.09	8.17–13.15	12.93	9.78–19.31	16.06	10.43–34.91
	50	9.73	8.01–12.51	12.28	9.42–17.50	14.98	10.20–30.03
	20	9.22	7.72–11.53	11.41	8.99–15.61	13.59	9.74–24.07
	10	8.81	7.47–10.83	10.75	8.69–14.30	12.56	9.37–20.67
	5	8.39	7.22–10.09	10.07	8.30–12.97	11.54	8.89–17.51
	2	7.79	6.81–9.15	9.15	7.73–11.32	10.24	8.35–14.23
	1	7.31	6.48–8.41	8.44	7.34–10.10	9.28	7.85 -11.99
41049	100	6.69	5.64–8.32	7.14	5.92–9.35	7.63	5.98–12.71
	50	6.53	5.57–8.02	6.96	5.84–8.89	7.48	5.93–11.75
	20	6.30	5.45–7.59	6.70	5.69–8.31	7.25	5.88–10.73
	10	6.11	5.35–7.27	6.48	5.57–7.88	7.05	5.83–9.94
	5	5.91	5.22–6.87	6.25	5.46–7.49	6.82	5.75–9.14
	2	5.61	5.02–6.41	5.91	5.24–6.88	6.48	5.61–8.19
	1	5.36	4.85–6.03	5.63	5.06–6.44	6.18	5.49–7.43
SIMAR-44	100	4.49	4.31–4.70	6.84	6.37–7.41	10.68	9.39–12.36
	50	4.43	4.25–4.63	6.64	6.20–7.16	10.03	8.96–11.51
	20	4.34	4.18–4.52	6.35	5.97–6.79	9.19	8.31–10.32
	10	4.26	4.11–4.42	6.12	5.78–6.51	8.56	7.84–9.50
	5	4.17	4.03–4.32	5.87	5.57–6.20	7.94	7.36–8.69
	2	4.02	3.90–4.16	5.51	5.26–5.78	7.14	6.70–7.69
	1	3.90	3.79–4.02	5.22	5.01–5.44	6.54	6.20–6.94

## Conclusions

This paper proposes a novel methodology for wave height time series modelling based on the assumption that, given a time series where the high waves are less common than lower ones, its distribution can be modelled as a mixture of a normal distribution with a uniform distribution. The methodology is based on the method of moments, and we use it to establish the threshold for the distribution estimation of the values over a peak methodology (POT). The automatic determination of this threshold is an important task, given that the alternative is to use a trial and error method which, as several authors agree, can be problematic and quite subjective. The whole approach is tested on nine real-world time series collected from the Gulf of Alaska (46001 and 46075), from Puerto Rico (41043, 41044, 41046, 41047, 41048 and 41049), and from Spain (SIMAR-44). For SIMAR-44, we compare our return periods with those obtained by Mazas and Hamm. The return periods obtained for the rest buoys can be considered as an initial approximation given the reduced length of the time series.

The experimentation is divided into three stages: the first verifies that the time series do not follow a normal distribution and that it, therefore, makes sense to apply the proposed methodology. The second one analysed the estimation of the distribution in the nine time series, showing that the estimated theoretical distribution fits the empirical one. These results are corroborated by a Kolmogorov-Smirnov test where $Z < Q(1-\alpha )$ in all databases. For the third experiment, we use the percentiles 95%, 97.5% and 99% of the estimated theoretical distribution as possible thresholds for the POT distribution estimation. Results show that the best-fitted distribution for the POT is the Generalized Pareto Distribution in all cases, showing their return periods and confidence intervals.

A future line of work could approach the segmentation of the time series based on the percentiles of the obtained distribution and perform a posterior prediction of the segments obtained. We also plan to extend this work using time series from different fields and more advanced methods for forecasting, such as artificial neural networks. One line of work already underway is eliminating uniform noise, after which the extraction of extreme values can be carried out on a normal distribution. Although the probability distributions of extreme values are independent from the starting distribution, we believe that knowledge about them would allow a better approximation.

## Data Availability

The datasets generated and/or analysed during the current study and the code generated in the experimental design are available at https://github.com/amduran/mixed_distributions.git, with the exception of SIMAR-44 which is available on request from Puertos del Estado.

## References

[1] Peng S (2019). Improving the real-time marine forecasting of the northern south china sea by assimilation of glider-observed t/s profiles. Sci. Rep..

[2] Soares CG, Scotto M (2001). Modelling uncertainty in long-term predictions of significant wave height. Ocean Eng..

[3] Saetra, Ø. & Bidlot, J.-R. *Assessment of the ECMWF Ensemble Prediction Sytem for Waves and Marine Winds* (European Centre for Medium-Range Weather Forecasts, 2002).

[4] Feng X, Tsimplis M, Yelland M, Quartly G (2014). Changes in significant and maximum wave heights in the norwegian sea. Global Planet. Change.

[5] Esling P, Agon C (2012). Time-series data mining. ACM Comput. Surv. (CSUR).

[6] Fontes CH, Budman H (2017). A hybrid clustering approach for multivariate time series-a case study applied to failure analysis in a gas turbine. ISA Trans..

[7] Pérez-Ortiz M (2017). On the use of evolutionary time series analysis for segmenting paleoclimate data. Neurocomputing.

[8] Kim J-S, Seo K-W, Chen J, Wilson C (2022). Uncertainty in grace/grace-follow on global ocean mass change estimates due to mis-modeled glacial isostatic adjustment and geocenter motion. Sci. Rep..

[9] Omranian N, Mueller-Roeber B, Nikoloski Z (2015). Segmentation of biological multivariate time-series data. Sci. Rep..

[10] Bagnall A, Lines J, Hills J, Bostrom A (2015). Time-series classification with COTE: The collective of transformation-based ensembles. IEEE Trans. Knowl. Data Eng..

[11] Nikolaou A (2015). Detection of early warning signals in paleoclimate data using a genetic time series segmentation algorithm. Clim. Dyn..

[12] Zhao Y (2016). A novel bidirectional mechanism based on time series model for wind power forecasting. Appl. Energy.

[13] Mathiesen M (1994). Recommended practice for extreme wave analysis. J. Hydraul. Res..

[14] Coles, S., Bawa, J., Trenner, L. & Dorazio, P. *An Introduction to Statistical Modeling of Extreme Values*, vol. 208 (Springer, 2001).

[15] Méndez FJ, Menéndez M, Luceño A, Losada IJ (2006). Estimation of the long-term variability of extreme significant wave height using a time-dependent peak over threshold (pot) model. J. Geophys. Res.: Oceans.

[16] Mazas F, Hamm L (2011). A multi-distribution approach to pot methods for determining extreme wave heights. Coast. Eng..

[17] Schwarz G (1978). Estimating the dimension of a model. Ann. Stat..

[18] Akaike, H. Information theory and an extension of the maximum likelihood principle. In *Selected Papers of Hirotugu Akaike* 199–213 (Springer, 1998).

[19] Petrov V, Soares CG, Gotovac H (2013). Prediction of extreme significant wave heights using maximum entropy. Coast. Eng..

[20] Durán-Rosal A, Fernández J, Gutiérrez P, Hervás-Martínez C (2017). Detection and prediction of segments containing extreme significant wave heights. Ocean Eng..

[21] Dorado-Moreno M (2017). Robust estimation of wind power ramp events with reservoir computing. Renew. Energy.

[22] Guijo-Rubio D (2018). Prediction of low-visibility events due to fog using ordinal classification. Atmos. Res..

[23] Durán-Rosal A (2018). Efficient fog prediction with multi-objective evolutionary neural networks. Appl. Soft Comput..

[24] Bowman K, Shenton L (2004). Estimation: Method of moments. Encycl. Stat. Sci..

[25] Jonathan P, Ewans K (2013). Statistical modelling of extreme ocean environments for marine design: A review. Ocean Eng..

[26] Mackay E, Johanning L (2018). Long-term distributions of individual wave and crest heights. Ocean Eng..

[27] DeLeo F, Besio G, Briganti R, Vanem E (2021). Non-stationary extreme value analysis of sea states based on linear trends analysis of annual maxima series of significant wave height and peak period in the mediterranean sea. Coast. Eng..

[28] Davison AC, Smith RL (1990). Models for exceedances over high thresholds. J. R. Stat. Soc. Ser. B (Methodol.).

[29] Ferreira J, Soares CG (1998). An application of the peaks over threshold method to predict extremes of significant wave height. J. Offshore Mech. Arct. Eng..

[30] Caires S, Sterl A (2005). 100-year return value estimates for ocean wind speed and significant wave height from the era-40 data. J. Clim..

[31] Stefanakos CN, Athanassoulis GA (2006). Extreme value predictions based on nonstationary time series of wave data. Environmetrics.

[32] Jonathan P, Randell D, Wadsworth J, Tawn J (2021). Uncertainties in return values from extreme value analysis of peaks over threshold using the generalised pareto distribution. Ocean Eng..

[33] Panchang VG, Gupta RC (1989). On the determination of three-parameter weibull mle’s. Commun. Stat.-Simul. Comput..

[34] Goda, Y. *Random Seas and Design of Maritime Structures*, vol. 33 (World Scientific Publishing Company, 2010).

[35] Clauset A, Shalizi CR, Newman ME (2009). Power-law distributions in empirical data. SIAM Rev..

[36] Wasserman, L. *All of Statistics: A Concise Course in Statistical Inference*, vol. 26 (Springer, 2004).

[37] White EP, Enquist BJ, Green JL (2008). On estimating the exponent of power-law frequency distributions. Ecology.

[38] Bauke H (2007). Parameter estimation for power-law distributions by maximum likelihood methods. Eur. Phys. J. B.

[39] Hosking JR, Wallis JR (1987). Parameter and quantile estimation for the generalized pareto distribution. Technometrics.

[40] Deluca A, Corral Á (2013). Fitting and goodness-of-fit test of non-truncated and truncated power-law distributions. Acta Geophys..

[41] Kang S, Song J (2017). Parameter and quantile estimation for the generalized pareto distribution in peaks over threshold framework. J. Korean Stat. Soc..

[42] Chakravarty, I. M., Roy, J. & Laha, R. G. *Handbook of Methods of Applied Statistics* (McGraw-Hill, 1967).

[43] Pearson, E. S. & Hartley, H. O. *Biometrika Tables for Statisticians* (Cambridge University Press, 1966).

[44] Pickands J (1975). Statistical inference using extreme order statistics. Ann. Stat..

[45] National buoy data center. http://www.ndbc.noaa.gov/. (National Oceanic and Atmospheric Administration of the USA (NOAA), 2021).

